# Intergenerational transmission of maternal childhood maltreatment, prenatal substance exposure, and internalizing and externalizing symptoms in early adolescence at age 12

**DOI:** 10.1111/jcpp.70030

**Published:** 2025-08-12

**Authors:** Meeyoung O. Min, June‐Yung Kim, Sonia Minnes, Rosa Kim, Lynn T. Singer

**Affiliations:** ^1^ College of Social Work University of Utah Salt Lake City UT USA; ^2^ Department of Social Work, College of Nursing and Professional Disciplines University of North Dakota Grand Forks ND USA; ^3^ Jack, Joseph and Morton Mandel School of Applied Social Sciences Case Western Reserve University Cleveland OH USA; ^4^ Department of Population and Quantitative Health Sciences, School of Medicine Case Western Reserve University Cleveland OH USA

**Keywords:** Intergenerational transmission of maltreatment, prenatal substance exposure, internalizing symptoms, externalizing symptoms, early adolescence

## Abstract

**Background:**

Few studies have examined the intergenerational impact of maternal childhood maltreatment (MCM) in the context of prenatal substance exposure (PSE). This study investigates whether PSE is part of the pathway of MCM or an independent risk factor affecting offspring psychopathology.

**Methods:**

Participants were 284 birth mother–child (44% male) dyads, primarily Black, low‐income, enrolled at birth. Exposure to alcohol, tobacco, marijuana, or cocaine in utero was assessed at 1 month postpartum. MCM was assessed at child age 4, and maternal psychological distress and offspring maltreatment at child age 10. Mother‐ and child‐reported internalizing and externalizing symptoms were assessed at child age 12 using the Child Behavior Checklist and the Youth Self‐Report. Structural equation modeling was conducted to test sequential mediation pathways examining the direct and indirect associations of MCM with child internalizing and externalizing symptoms via PSE, maternal psychological distress, and offspring maltreatment, adjusting for covariates.

**Results:**

MCM was directly related to adolescent‐reported internalizing and externalizing symptoms, whereas PSE was directly related to adolescent‐reported externalizing symptoms. MCM and PSE were indirectly related to mother‐reported internalizing and externalizing symptoms via maternal psychological distress. Only PSE was related to offspring maltreatment at 10 years, and offspring maltreatment was related to both mother‐ and adolescent‐reported internalizing and externalizing symptoms at 12 years.

**Conclusions:**

MCM and PSE may increase offspring vulnerability to psychopathology, highlighting the importance of evaluating historical risks that mothers may transmit from their own childhood maltreatment and prenatal substance use in assessing offspring psychopathology.

## Introduction

Child maltreatment is a pervasive and serious public health problem. In the United States, over 3 million children were subjects of child maltreatment reports in 2023; of those, 16% had evidence of maltreatment (US Department of Health & Human Services, 2025). It is estimated that, before age 18, more than one third of all US children (37.4%) are the subjects of investigated child maltreatment reports (Kim, Wildeman, Jonson‐Reid, & Drake, 2017), and 1 in 8 of those reported (12.5%) are substantiated by Child Protective Services (Wildeman et al., 2014).

While the pernicious impact of child maltreatment has been extensively documented across multiple domains of mental (Danese & Widom, 2023; McLaughlin, Colich, Rodman, & Weissman, 2020; Min, Farkas, Minnes, & Singer, 2007) and physical health (Grummitt et al., 2021; Min, Minnes, Kim, & Singer, 2013), its effects may also cut across generations (e.g., Buss et al., 2017; Min, Singer, Minnes, Kim, & Short, 2013; Moog et al., 2023; Uy et al., 2023). Children of mothers who had themselves experienced childhood maltreatment are at elevated risk of exposure to higher maternal psychological distress (Harris et al., 2023; Humphreys et al., 2020; Minnes et al., 2008) and harsh discipline and maltreatment (Madigan et al., 2019; Plant, Jones, Pariante, & Pawlby, 2017), contributing to emotional and behavioral problems (Kim et al., 2024; Ma et al., 2022; Negriff, Palmer Molina, & Hackman, 2020). However, few studies have examined the intergenerational impact of maternal childhood maltreatment (MCM) in the context of prenatal substance use, despite their well‐established association (Elliott et al., 2014; Racine, McDonald, Chaput, Tough, & Madigan, 2020). Only one cross‐sectional study to date (Warmingham et al., 2024) has examined both MCM and prenatal substance (alcohol, tobacco, marijuana, and cocaine) exposure (PSE). That study reported that both MCM and PSE were associated with offspring maltreatment, which was associated with greater mother‐reported externalizing symptoms in adolescents. MCM was directly related to mother‐reported internalizing symptoms. However, this study was limited to girls, with less than 10% of mothers reporting prenatal substance use. Explicating the roles of PSE and the postnatal developmental environment as intergenerational risk processes of MCM is crucial for effective interventions and maximizing benefits for children.

### Maternal childhood maltreatment, PSE, and risk processes to offspring psychopathology

Childhood maltreatment alters the structure and function of stress‐susceptible brain regions, such as the hypothalamic–pituitary–adrenal axis, affecting afflicted individuals' ability to regulate stress and emotion (Teicher, Samson, Anderson, & Ohashi, 2016); thus, increasing the risk of developing psychopathology and health‐compromising behaviors including substance use (Hughes et al., 2017; Norman et al., 2012; Rogers et al., 2022). Childhood maltreatment has been associated with an earlier age of onset of alcohol and drug use (Hamburger, Leeb, & Swahn, 2008; Kerr et al., 2009), daily tobacco smoking at ages 22–24 (Topitzes, Mersky, & Reynolds, 2010), and daily cannabis use and dependence at age 21 (Mills, Kisely, Alati, Strathearn, & Najman, 2017). These substance‐using behaviors may continue during pregnancy (Chung et al., 2010; Towers et al., 2020). The 2023 National Survey on Drug Use and Health estimated that 9.4% of pregnant women used tobacco, 8.4% used alcohol, and 4.9% used illicit drugs including marijuana, opioids, and cocaine during the past month, with polysubstance use common among illicit drug‐using women (Substance Abuse and Mental Health Services Administration, 2024).

PSE interferes with fetal brain development by altering neurotransmitter systems in the prefrontal cortex, affecting emotional and behavioral arousal and regulation and stress response (Ross, Graham, Money, & Stanwood, 2015), which may increase vulnerability to psychopathology (Eiden, Perry, Ivanova, & Marcus, 2023). Mounting evidence, although mixed, indicates a link between PSE and the development of psychopathology. Greater prenatal alcohol exposure was related to internalizing and externalizing symptoms in a meta‐analysis of six longitudinal cohorts (Jacobson et al., 2021); prenatal tobacco exposure was associated with suboptimal developmental trajectories of internalizing symptoms in poly‐drug exposed children (Min et al., 2021) and with conduct problems using meta‐analyses of 25 studies (Ruisch, Dietrich, Glennon, Buitelaar, & Hoekstra, 2018); prenatal marijuana/cannabis exposure was associated with more aggressive behavior at age 5 (Keim et al., 2024) and increased teacher‐ and child‐reported externalizing symptoms at ages 7–9 in a population‐based birth cohort (El Marroun et al., 2019); and prenatal cocaine exposure was related to teacher‐ and caregiver‐rated externalizing symptoms at ages 7, 9, 11, and 13 years (Bada et al., 2011) and to adolescents' self‐rated externalizing symptoms at ages 12 and 15 years (Min, Minnes, Yoon, Short, & Singer, 2014). It is important to note that the effects of PSE are highly variable depending on the specific dosage, chronicity, gestational timing of the exposure, and co‐exposure to other substances (Eiden et al., 2023), contributing to the inconsistent findings.

Continuing substance use post pregnancy can also compromise parenting ability (Hatzis, Dawe, Harnett, & Loxton, 2019). Substance use may offset reward systems in the brain, making parent–child interaction less rewarding and pleasant (Rutherford et al., 2021). Maternal psychological functioning has been considered a main mechanism connecting MCM with children's behavior (Cooke, Racine, Plamondon, Tough, & Madigan, 2019). Maternal psychological distress may contribute to caregivers' emotional unavailability, to a lack of consistent sensitive responses to children's needs, and to the increased likelihood of coercive, maltreating parenting behaviors, elevating the risk for psychopathology in their children (Goodman et al., 2011; Stith et al., 2009). Multiple meta‐analyses have demonstrated that maternal psychological distress mediates the associations of MCM with offspring psychopathology (Loheide‐Niesmann, Riem, & Cima, 2024; Zhang, Mersky, Gruber, & Kim, 2023). Emerging evidence also suggests offspring maltreatment as a pathway linking MCM with offspring internalizing and externalizing symptoms in childhood (Plant et al., 2017; Russotti, Warmingham, Handley, Rogosch, & Cicchetti, 2021) and in adolescence (Kızıltepe & Irmak, 2024; Warmingham et al., 2024).

### The current study

The current study examined associations among MCM, PSE, maternal psychological functioning, and adolescents' internalizing and externalizing symptoms in an urban low‐income birth cohort of boys and girls. We modeled intergenerational risk processes that may elucidate pathways from MCM to offspring psychopathology, while specifying PSE as a potential mediator of MCM, directly and indirectly associated with offspring psychopathology through maternal psychological distress and parenting behavior. Our study examined the role of PSE in the context of intergenerational transmission of MCM, testing whether PSE is part of the pathway of MCM or an independent risk factor that increases child vulnerability to psychopathology. We utilized both maternal and adolescent reports to assess internalizing and externalizing symptoms more comprehensively given the well‐documented discrepancy across informants (De Los Reyes et al., 2015; Khoury et al., 2022). Relevant confounders of MCM and adolescents' psychopathology were assessed, including maternal education (Min et al., 2007), child sex (Endendijk et al., 2017), and quality of home environment and ecological resources as markers characterizing the socio‐economic developmental context (Min et al., 2024; St‐Laurent, Dubois‐Comtois, Milot, & Cantinotti, 2019). We hypothesized that (1) more severe MCM would be associated with higher levels of PSE, (2) both MCM and PSE would be associated with increased maternal psychological distress and maltreating parenting behavior, and (3) these maternal factors would be related to greater internalizing and externalizing symptoms in their offspring.

## Methods

### Participants

The present study included 284 birth mother–child dyads from a birth cohort recruited between September 1994 and June 1996 at an urban teaching hospital in the Midwest USA for a prospective study on the developmental effects of prenatal cocaine exposure (Minnes et al., 2014; Singer, Albert, Minnes, Min, & Kim, 2024). Pregnant women at high risk for drug use (e.g., lack of prenatal care, behaviors suggesting intoxication, self‐admitted drug use) were given drug toxicology screenings per hospital policy. Mothers with severe mood disorders or schizophrenia, HIV positive status, chronic medical illness, or low intellectual functioning indicated in medical charts were excluded, as were infants with Down syndrome, fetal alcohol syndrome, or congenital heart defects. A total of 404 birth mothers and their newborns were enrolled at birth. Of the 404 mothers, 302 mothers were assessed for childhood maltreatment at 4 years postpartum, and 12 children died by the 12‐year assessment. The present study utilizes data from 284 birth mother–child dyads with complete data on MCM, PSE, and child behavioral assessment at age 12 years. Compared to the 120 mothers who were not included, the 284 participating mothers were more likely to have prenatal care visits (76% vs. 89%) and less likely to smoke cigarettes (75% vs. 62%) and to use cocaine (64% vs. 47%) during pregnancy; and their children were less likely to have low birth weight (<2,500 g; 35% vs. 23%), all *p*'s ≤ .01. No other differences were found.

The 284 mothers and their biological children (130 boys, 154 girls) were primarily Black (*n* = 235, 83%) with low socioeconomic status (SES; *n* = 277, 98%). At delivery, the mean (*SD*) maternal age was 27.6 (5.30), range 18–41, with 13% (*n* = 38) being married. Thirty‐nine percent of mothers (*n* = 112) had not finished high school, with a mean (*SD*) of 11.74 (1.54) years of education. About one‐third of mothers (*n* = 95) reported sexual abuse; 29% (*n* = 81) physical abuse, 23% (*n* = 64) emotional abuse, 30% (*n* = 86) emotional neglect, and 28% (*n* = 80) physical neglect. More than half (*n* = 157) reported at least one type of maltreatment, 37% (*n* = 106) more than one type of maltreatment, and 7% (*n* = 13) all five types of maltreatment. Urine, infant meconium analyses, or self‐report indicated that 243 (85%) mothers used at least one substance during pregnancy: 134 (47%) used cocaine, 171 (60%) cigarettes, 212 (75%) alcohol, and 81 (28%) marijuana. Two‐thirds of the mothers (*n* = 186) used two or more substances during pregnancy, including most (96%) mothers who used cocaine.

### Procedure

The Institutional Review Board of the participating hospital approved the study. The initial screenings and enrollment were conducted in the hospital. The follow‐up assessments were conducted at the university‐based research lab at 1, 6, and 12 months and 2, 4, 6, 9, 10, 11, and 12 years of age postpartum by separate examiners blind to PSE. Data in the present study were taken from assessments conducted when the children were 1 month (PSE), 4 (MCM), 10 (maternal psychological distress and offspring maltreatment), and 12 years of age (offspring psychopathology). Parental written informed consent was collected at each visit, with child assent beginning at age 9. A Certificate of Confidentiality (DA‐98‐91), exempting the study from legislative, judicial, or administrative attempts to obtain confidential information, was obtained from the Department of Health and Human Services. All participants were compensated with a monetary stipend, lunch, and/or transportation costs.

### Measures

#### Maternal childhood maltreatment (MCM)

Maternal childhood abuse and neglect were assessed using the Childhood Trauma Questionnaire (CTQ; Bernstein & Fink, 1998), a 28‐item self‐report inventory of emotional, physical, and sexual abuse, and emotional and physical neglect. Items were rated on a 5‐point scale according to frequency (*1 = never true* to *5 = very often true*) and summed to yield a total score for each maltreatment, with higher scores indicating greater severity. For descriptive purposes, scores indicating moderate to severe levels of maltreatment (>12 in emotional abuse, >9 in physical abuse and neglect, >7 in sexual abuse, and >14 in emotional neglect; Bernstein & Fink, 1998) were used to dichotomize the CTQ subscales. MCM was specified as a latent variable with the five continuous subscales, with the measurement errors of the two observed neglect variables being correlated given a substantial correlation between them.

#### Prenatal substance exposure (PSE)

At the newborn visit, birth mothers reported the amount and frequency of drug use for the month prior to and for each trimester of pregnancy using a Timeline Follow Back method (Singer et al., 2002). For cocaine use, the number of rocks consumed and the amount of money spent on cocaine per day were recalled and computed to a standard unit of cocaine, equivalent to $20 worth of crack cocaine. The number of drinks of beer, wine, or hard liquor per week, with each drink equivalent to 0.5 oz. of absolute alcohol, was recorded. The number of tobacco cigarettes and marijuana joints smoked per day was collected. For each drug, the frequency of use was rated on a Likert‐type scale (0 = *not at all to* 7 = *daily use*) to indicate the average number of days per week of drug use, except for cigarettes computed as the number smoked per day. PSE was specified as a latent variable with four indicators reflecting the amount of alcohol, tobacco, marijuana, and cocaine used due to substantial correlations between them.

#### Maternal psychological distress

At the 10‐year visit, maternal psychological distress was assessed with the Brief Symptom Inventory (Derogatis, 1992), a widely used 53‐item self‐report standardized assessment of psychological symptoms experienced in the past 7 days such as depression, anxiety, and hostility. Items were rated on a 5‐point scale, with higher scores indicating greater distress. The global severity index (GSI), the average rating of all 53 items, was used.

#### Maternal offspring maltreatment

At the 10‐year visit, offspring maltreatment was assessed with the Parent–Child Conflict Tactics Scale (PCCTS; Straus, Hamby, Finkelhor, Moore, & Runyan, 1998), a parent‐report on parenting behaviors related to child maltreatment. The psychological aggression scale assesses verbal and emotional abuse (5 items, e.g., “shouted, yelled, screamed”). The physical assault scale includes corporal punishment (5 items, e.g., “spanked on the bottom with a bare hand”), severe assault (4 items, e.g., “Threw or knocked down”), and very severe assault (4 item, e.g., “grab around the neck and choked”). Items were rated on a 5‐point scale according to their frequency, 0 = *never*, 1 = *once or twice in the past year*, 2 = *3–10 times in the past year*, 3 = *11 or more times in the past year*, 4 = *not in the past year*, *but it happened before*, which was recoded to: 0 = *never*, 1 = *not in the past year*, *but it happened before*, 2 = *once or twice in the past year*, 6 = *3–10 times in the past year*, 15 = *11 or more times in the past year*, using the midpoint for the original category 2 and 3 (Straus et al., 1998). The total score of physical assaults was calculated using weights to account for the severity by multiplying 2 for the severe assault and 3 for very severe assault (Straus et al., 1998). Higher scores indicate greater maltreatment. It was specified as a latent variable with the two subscales.

#### Offspring internalizing and externalizing symptoms

At the 12‐year visit, child behaviors were assessed using the Child Behavior Checklist for ages 6–18 (CBCL) and the Youth Self‐Report (YSR; Achenbach & Rescorla, 2001). The CBCL is a 112‐item parent rating, and the YSR is a 105‐item child self‐rating, both designed to assess emotional, behavioral, and social problems of children in the last 6 months. Standardized *T‐*scores of internalizing (i.e., withdrawn, somatic complaints, and anxious/depressed) and externalizing (i.e., aggression and delinquency) behaviors were used, with higher scores indicating greater symptoms.

#### Covariates

Maternal (age at delivery, race, and education) and child characteristics (sex, race, gestational age, and birth weight) were retrieved from hospital birth records and verified at the 1‐month postpartum visit. SES was assessed using the Hollingshead Two‐Factor Index (Hollingshead, 1971) at the 1‐month postpartum visit. The quality of the caregiving environment was assessed at age 9 via the caregiver report using the Home Observation of the Environment (Caldwell & Bradley, 1979). Ecological resources and support were assessed at age 12 using the External Assets subscale of the Developmental Assets Profile (Search Institute, 2005), a youth self‐report (ages 12–18) with a possible range of 0–30. Higher scores indicate greater assets.

### Data analysis

Data positively skewed were normalized using a log transformation (the four indicators of PSE and GSI) or square root transformation (physical aggression). Distributional characteristics were reported by the variables' original distribution, with normalized data used in bivariate and multivariate analyses. Bivariate associations between study variables were assessed using zero‐order Pearson correlations.

Structural equation modeling was conducted using Mplus 8.4 to evaluate sequential mediation pathways examining the direct and indirect associations of MCM and PSE with child internalizing and externalizing symptoms at age 12. To account for commonalities among the endogenous variables representing maternal functioning (maternal psychological distress and maltreating parenting behavior) and child psychopathology (maternal and adolescent reports), all within‐time cross‐domain correlations were specified. Direct paths from MCM and PSE to offspring internalizing and externalizing symptoms were specified to indicate other unmeasured mediators of MCM and PSE. The maximum likelihood method was used to fit the models. Model fit was examined using the chi‐square (*χ* ^2^) goodness‐of‐fit test, the Comparative Fit Index (CFI), Tucker–Lewis Index (TLI), root mean square error of approximation (RMSEA), and standardized root mean square residual (SRMR) indices. Values ≥.95 for CFI and TLI, ≤.06 for RMSEA, and ≤.08 for SRMR indicate a good fit (Hu & Bentler, 1998). Missing data on maternal psychological distress and parenting behavior (~21%) were estimated using full information maximum likelihood, allowing use of all available information from the observed data (Enders & Bandalos, 2001). With no missing covariates, the analytical model was based on all 284 birth mother–child dyads. The significance of the indirect association was tested using a bootstrap method based on 1,000 resamples (Preacher & Hayes, 2008). Model comparisons were conducted using the *χ* ^2^ difference (Δ*χ* ^2^) test (Kline, 1998). Two‐sided *p <* .05 indicated statistical significance. For ease of interpretation, parameter estimates are presented in standardized form.

## Results

Table 1 provides descriptive statistics, internal consistency (*α*), and bivariate correlations between key observed variables. Maternal and adolescent ratings of internalizing and externalizing symptoms were only moderately correlated (*r* = .25 on internalizing and .34 on externalizing), supporting our analytical decision not to aggregate the maternal and adolescent ratings but to examine them separately.

**Table 1:** Descriptive statistics and correlations (*r*) among key observed variables in the path model

	Maternal													Child					
	1	2	3	4	5	6	7	8	9	10	11	12	13	14	15	16	17	18	19
1. Maternal education	–	**−.19**	−.08	−.10	**−.24**	**−.21**	**−.12**	**−.21**	−.07	**−.23**	−.11	.08	.12	.09	**−.14**	−.02	−.09	.00	−.06
Maternal CTQ																			
2. Emotional abuse		–	.**74**	.**52**	.**61**	.**52**	.06	.**18**	.**15**	.09	.**32**	.11	.10	−.01	.02	.**21**	.**20**	.**12**	.**15**
3. Physical abuse			–	.**52**	.**49**	.**50**	.07	.**14**	.**12**	.06	.**19**	.12	.**15**	−.04	−.00	.**15**	.**12**	.**12**	.**15**
4. Sexual abuse				–	.**36**	.**40**	.03	.**13**	.11	.04	.**22**	.08	.**14**	−.04	−.06	.**20**	.**23**	.10	.**13**
5. Emotional neglect					–	.**62**	.05	.11	.05	.10	.**22**	.04	.08	−.02	−.01	.**15**	.**21**	.07	.11
6. Physical neglect						–	−.02	.10	.**14**	.04	.09	−.06	−.04	.00	.02	.04	.04	.03	.06
PSE																			
7. Alcohol^a^							–	.**48**	.**15**	.**55**	.**19**	.12	.12	−.07	−.05	−.00	.10	.08	.**14**
8. Tobacco^a^								–	.**25**	.**47**	.**24**	.06	.04	−.09	.05	.05	.**15**	−.03	.10
9. Marijuana^a^									–	.**16**	.11	.04	.05	.00	.03	.07	−.05	.03	.**12**
10. Cocaine^a^										–	.**19**	.**16**	.06	−.06	−.05	.02	.**14**	−.02	.**18**
11. Psychological distress^a^											–	.**39**	.**27**	−.00	.04	.**45**	.**39**	.13	.10
PCCTS																			
12. Psychological												–	.**69**	.**17**	−.12	.**27**	.**36**	.**14**	.**19**
13. Physical^b^													–	.13	−.01	.**17**	.**27**	.14	.12
14. Child sex														–	**−.12**	.10	.11	.**16**	.10
15. DAP external assets															–	−.09	**−.13**	**−.29**	.**37**
16. CBCL internalizing																–	.**66**	.**25**	.**12**
17. CBCL externalizing																	–	.**22**	.**34**
18. YSR internalizing																		–	.**54**
19. YSR externalizing																			–
Mean (*n*)	11.74	9.45	8.75	8.89	11.73	8.25	5.83	7.52	0.97	9.90	0.36	11.97	5.37	(130)	21.37	48.00	49.96	53.18	49.04
*SD* (%)	1.54	5.08	4.80	6.20	5.49	3.99	14.61	10.42	3.65	26.87	0.46	10.95	7.96	(45.8)	4.49	10.68	12.18	9.93	10.16
*α*		.85	.84	.94	.85	.70	–	–	–	–	.95	.68	.69	–	.90	.88	.93	.86	.87

Bold indicates significance at *p* < .05; Skewed data were normalized for bivariate and multivariate analyses using ^a^log transformation or ^b^square root transformation, with raw data reported for mean and *SD*. CBCL, Child Behavioral Checklist; CTQ, Childhood Trauma Questionnaire; DAP, Developmental Asset Profile; PCCTS, Parent–Child Conflict Tactic Scale; PSE, Prenatal Substance Exposure; YSR, Youth Self‐Report.

### Model estimation

**Figure 1:**
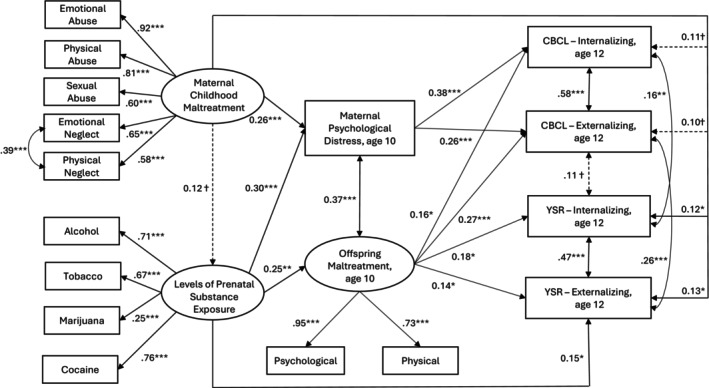
The association of maternal childhood maltreatment with offspring internalizing and externalizing symptoms at age 12. *χ* ^2^ (126) = 167.62, *p* = .008, CFI = 0.972, TLI = 0.963, RMSEA = 0.034 (90% CI = 0.018–0.047), SRMR = 0.047. CBCL, Child Behavior Checklist; YSR, Youth Self‐Report. Rectangles indicate observed variables, and ovals represent latent constructs. All path coefficients are standardized. Single‐arrowed lines represent standardized path coefficients; double‐arrowed lines represent correlations. Solid lines indicate significant (*p* ≤ .05) coefficients; dotted lines indicate non‐significant (*p* > .05) coefficients. Only path coefficients with *p* < .10 are shown; covariates and errors associated with the measurement model are not displayed for parsimony. Estimates were adjusted for maternal education on maternal childhood maltreatment and prenatal substance exposure, child sex on offspring maltreatment, and external assets on YSR internalizing and externalizing symptoms. MCM was indirectly, via psychological distress, related to mother‐reported internalizing (indirect effect of MCM, *β* = .098, *SE* = .035, *p* = .005) and externalizing symptoms (indirect effect of MCM, *β* = .066, *SE* = .027, *p* = .012). PSE was indirectly, via maternal psychological distress, related to mother‐reported internalizing (indirect effect of PSE, *β* = .113, *SE* = .045, *p* = .012) and externalizing symptoms (indirect effect of PSE, *β* = .077, *SE* = .035, *p* = .027). The final model accounted for 23% and 24% of the variation in mother‐reported internalizing and externalizing symptoms, respectively, and 12% and 20% in adolescent‐reported internalizing and externalizing symptoms at age 12, respectively. ^†^ *p* < .10, *p ≤ .05, **p ≤ .01, ***p ≤ .001

The initial, saturated model included: (1) paths from MCM to all three mediators and the four indicators of mother‐ and adolescent‐reported internalizing and externalizing symptoms at age 12; (2) paths from PSE to the other two mediators and the four indicators of internalizing and externalizing symptoms; (3) paths from maternal psychological distress and offspring maltreatment to the four indicators of internalizing and externalizing symptoms; (4) correlations between maternal psychological distress and offspring maltreatment at age 10; (5) correlations ndbetween mother‐ and adolescent‐reported internalizing and externalizing symptoms; and (6) correlations between all exogenous (i.e., MCM and all covariates) variables. This initial model produced a good model fit, *χ* ^2^ (111) = 152.61, *p* = .005, CFI = 0.972, TLI = 0.958, RMSEA = 0.036 (90% CI = 0.020–0.050), SRMR = 0.044. To achieve a more parsimonious model, path coefficients with a significance level of *p ≥*.15 were set to zero (Hosmer, Lemeshow, & Sturdivant, 2013), *χ* ^2^ (126) = 167.62, *p* = .008, CFI = 0.972, TLI = 0.963, RMSEA = 0.034 (90% CI = 0.018–0.047), SRMR = 0.047, yielding an insignificant difference in model fit, Δ*χ* ^2^ (15) = 15.01, *p* = .45. This reduced model was accepted as the final model (Figure 1).

Figure 1 indicates the least marginally significant (*p* < .10) path coefficients. Adjusting for relevant covariates, MCM was related to maternal psychological distress at age 10 (*β* = .26, *SE* = .070, *p* < .001) but was not related to PSE (*β* = .12, *SE* = .073, *p* = .099) nor offspring maltreatment at age 10 (*β* = .13, *SE* = .089, *p* = .135). Maternal psychological distress was related to mother‐reported internalizing (*β* = .38, *SE* = .081, *p* < .001) and externalizing symptoms at age 12 (*β* = .26, *SE* = .078, *p* = .001). MCM was indirectly, via psychological distress, related to mother‐reported internalizing (indirect effect of MCM, *β* = .098, *SE* = .035, *p* = .005) and externalizing symptoms (indirect effect of MCM, *β* = .066, *SE* = .027, *p* = .012). MCM was also directly related to adolescent‐reported internalizing (*β* = .12, *SE* = .057, *p* = .036) and externalizing symptoms at age 12 (*β* = .13, *SE* = .054, *p* = .014). PSE was related to maternal psychological distress (*β* = .30, *SE* = .084, *p* < .001) and offspring maltreatment (*β* = .25, *SE* = .098, *p* = .011). Similar to MCM, PSE was indirectly, via maternal psychological distress, related to mother‐reported internalizing (indirect effect of PSE, *β* = .113, *SE* = .045, *p* = .012) and externalizing symptoms (indirect effect of PSE, *β* = .077, *SE* = .035, *p* = .027). Although individual paths from PSE to offspring maltreatment at age 10 and from offspring maltreatment to mother‐ and adolescent‐reported internalizing and externalizing symptoms at age 12 were all significant, the indirect effect of PSE via offspring maltreatment did not reach significance (*p*'s ≥ .067). PSE was also directly related to higher adolescent‐reported externalizing symptoms (*β* = .15, *SE* = .062, *p* = .015). Offspring maltreatment was related to both mother‐ and adolescent‐reported internalizing (*β* = .16, *SE* = .082, *p* = .048 on the CBCL, *β* = .18, *SE* = .073, *p* = .015 on the YSR) and externalizing symptoms (*β* = .27, *SE* = .082, *p* = .001 on the CBCL, *β* = .14, *SE* = .068, *p* = .036 on the YSR). Maternal psychological distress and offspring maltreatment were correlated (*r* = .37, *p* < .001); the two indicators of internalizing symptoms were correlated (*r* = .16, *p* = .008) and so were the two indicators of externalizing symptoms (*r* = .26, *p* < .001). In terms of covariates, higher maternal education was related to both MCM (*r* = − .20, *p* < .001) and PSE (*β* = −.25, *SE* = .075, *p* = .001). Boys experienced greater maltreatment than girls (*β* = .174, *SE* = .067, *p* = .009). Greater external assets were related to fewer adolescent‐reported internalizing (*β* = −.26, *SE* = .057, *p* < .001) and externalizing symptoms (*β* = −.34, *SE* = .055, *p <* .001). Table 2 summarizes the direct and indirect associations of MCM, PSE, maternal psychological distress, and offspring maltreatment with offspring internalizing and externalizing symptoms.

**Table 2:** Decomposition of the standardized estimates of maternal childhood maltreatment on offspring psychopathology at age 12

	CBCL				YSR			
	Internalizing		Externalizing		Internalizing		Externalizing	
	*β* (*SE*)	*p*	*β* (*SE*)	*p*	*β* (*SE*)	*p*	*β* (*SE*)	*p*
MCM								
Direct	.107 (.057)	.063	.095 (.051)	.065	.119 (.057)	.036	.132 (.054)	.014
Indirect	.122 (.040)	.002	.124 (.039)	.002	.024 (.022)	.287	.041 (.023)	.069
Total	.229 (.062)	<.001	.219 (.055)	<.001	.142 (.055)	.010	.173 (.053)	.001
Levels of PSE								
Direct	−.128 (.079)	.102	.035 (.077)	.650	−.041 (.071)	.564	.150 (.062)	.015
Indirect	.154 (.053)	.004	.144 (.051)	.005	.044 (.027)	.101	.035 (.022)	.104
Total	.024 (.072)	.735	.179 (.073)	.014	.003 (.069)	.966	.185 (.064)	.004
Maternal psychological distress^a^								
Direct	.375 (.081)	<.001	.255 (.078)	.001	–	–	–	–
Indirect from MCM	.098 (.035)	.005	.066 (.027)	.012	–	–	–	–
Indirect from PSE	.113 (.045)	.012	.077 (.035)	.027	–	–	–	–
Indirect from MCM → PSE	.014 (.011)	.211	.009 (.008)	.242	–	–	–	–
Offspring maltreatment								
Direct	.163 (.082)	.048	.269 (.082)	.001	.177 (.073)	.015	.142 (.068)	.036
Indirect from MCM	.022 (.021)	.292	.036 (.027)	.181	.023 (.020)	.249	.019 (.016)	.250
Indirect from PSE	.040 (.028)	.146	.067 (.036)	.067	.044 (.101)	.101	.035 (.022)	.104
Indirect from MCM → PSE	.005 (.005)	.296	.008 (.007)	.238	.005 (.005)	.257	.004 (.004)	.270

CBCL, Child Behavior Checklist; MCM, maternal childhood maltreatment; PSE, prenatal substance exposure; YSR, Youth Self‐Report.

^a^ Direct paths from maternal psychological distress to internalizing symptoms and to externalizing symptoms on the YSR were removed in the final model due to the lack of significance (*p ≥* .15). Estimates were adjusted for maternal education on MCM and PSE, child sex on offspring maltreatment, and external assets on YSR internalizing and externalizing symptoms.

## Discussion

This study demonstrated that both MCM and PSE increased offspring vulnerability to psychopathology directly and indirectly in a low‐income urban sample of prenatal cocaine/polydrug‐using mothers and their children. However, PSE was not a pathway underlying an intergenerational risk process of MCM to offspring psychopathology. Instead, independent of MCM, PSE was directly related to higher adolescent‐reported externalizing symptoms even after maternal psychological distress and maltreating parenting behavior were considered, supporting the long‐lasting teratologic effects of PSE (Ross et al., 2015). MCM was related to greater internalizing and externalizing symptoms in offspring, directly when adolescents were informants but indirectly via maternal psychological distress when mothers were informants, demonstrating the intergenerational effect of MCM regardless of informant while suggesting pathways varied by informant. These core findings integrate two separate yet interrelated lines of inquiry toward a more nuanced understanding of the intergenerational risk process, where both the history of MCM and PSE are additively associated with increased risk for developing psychopathology in offspring. The present study is one of the few studies to document the role of PSE in the context of intergenerational transmission of MCM and, to our knowledge, the first using a prospective birth cohort of children of mothers with varying degrees of childhood maltreatment and prenatal substance use.

Contrary to our hypothesis, a non‐significant, yet marginal (*p* = .099) relationship was found between MCM and PSE. This could be due to the relative lack of variability in PSE in our sample where the majority reported some levels of PSE. Also, given that almost all mothers were categorized as low SES with limited educational obtainments, PSE might be highly confounded with poverty and its related psychosocial factors such as domestic violence and drug‐using partners and friends (Min, Tracy, & Park, 2014). Replication studies with a more balanced distribution in PSE may clarify the relationship of MCM with PSE.

In our model of multiple mediators with two markers reflecting maternal psychological functioning, only psychological distress was a significant mediator of both MCM and PSE on mother‐reported offspring outcomes. It underscores the well‐recognized pivotal impact of maternal psychological distress, regardless of its antecedents, that confers an increased risk of developing psychopathology in offspring even after accounting for maltreating parenting behaviors (e.g., Plant et al., 2017). Maternal offspring maltreatment was associated only with PSE, not with MCM, indicating that maltreatment was likely a function of PSE, rather than MCM, in this sample with high prevalence of both MCM and PSE. It is noteworthy that MCM was not related to PSE but was related to adolescent‐reported internalizing and externalizing symptoms with similar effect sizes (Cohen, 1992), a finding that warrants replication. Despite the relationships of maternal offspring maltreatment with both mother‐ and adolescent‐reported internalizing and externalizing symptoms in offspring, offspring maltreatment did not emerge as a mediator of PSE. Given the frequent co‐occurrence of maternal psychological distress and offspring maltreatment, our simultaneous analysis of multiple associated pathways suggests that the intergenerational transmission of MCM can be better explained by maternal psychological distress, highlighting maternal psychological distress as a priority for interventions aimed at counteracting the intergenerational transmission of risk.

The direct relationship of MCM with adolescent‐reported internalizing and externalizing symptoms may imply other intermediary risk mechanisms. Additional adverse life events (Fereidooni, Daniels, & Lommen, 2024), lack of social support (Bosquet Enlow, Englund, & Egeland, 2018), poor mother–child attachment (Alto et al., 2021), accumulations of such psychosocial risks (Racine et al., 2018), or epigenetic methylation (Monk, Spicer, & Champagne, 2012) may account for the direct relationship apparent in our study, areas of consideration in future studies.

The findings need to be considered in light of limitations. First, the generalizability of our findings is limited to the characteristics of our cohort, which is urban, low‐income, and predominantly Black adolescents, where about half of their mothers used cocaine and other substances during pregnancy. Second, MCM was constructed as a latent variable to capture high correlations among multiple types of abuse and neglect. Given that abuse versus neglect could be differentially associated with different developmental domains and thus, with later caregiving behaviors (e.g., abuse linked to aggression whereas neglect to health problems and cognitive abilities; Usacheva, Choe, Liu, Timmer, & Belsky, 2022), the observed associations of MCM with PSE and maternal psychological functioning could reflect abuse experience rather than neglect, a hypothesis that should be tested in future studies. Third, although the time reference of the CTQ guided mothers to recall their childhood, the temporal precedence of MCM (assessed at 4‐year postpartum) to PSE (assessed at childbirth) could be ambiguous. The use of a retrospective self‐report to assess MCM might have compromised its reliability due to potential memory errors or suppression (Rucklidge, Brown, Crawford, & Kaplan, 2006). Nonetheless, retrospective self‐reports of childhood maltreatment have been shown to be stronger correlates of psychosocial outcomes than objectively assessed measures (Danese & Widom, 2023; Latham et al., 2021). Fourth, the measure of offspring maltreatment overlooked neglect and might not fully capture the effects from MCM and PSE. We also relied on maternal reports assessing offspring maltreatment, subjective to social desirability bias. The insignificant relationship of MCM with offspring maltreatment could be due to the limited measurement of offspring maltreatment or/and mothers' tendency to underreport their maltreating behavior. Lastly, the significant indirect relationship of MCM via maternal psychological distress with maternal report of offspring internalizing and externalizing behaviors could be due to biases associated with a single informant through shared method variance as well as mothers' cognitive biases, as psychologically distressed mothers tend to perceive their childhood and their children's behavior more negatively (Najman et al., 2001). Yet, it is these maternal perceptions that shape maternal responses and parenting practice, and thus, offspring psychopathology.

The notable strength of the present study includes controlling for single‐source bias and shared method variance. By simultaneously assessing maternal and adolescent reports on offspring psychopathology, we disentangled informant‐specific pathways from MCM to offspring psychopathology. The use of the Timeline Follow Back method enhanced recall accuracy in assessing PSE (Robinson, Sobell, Sobell, & Leo, 2014). Adjusting for multiple prenatal and postnatal socioeconomic markers such as maternal education and ecological resources, often considered common etiological factors of MCM, PSE, and offspring psychopathology (Belsky, 1993; Dixon, Browne, & Hamilton‐Giachritsis, 2009), increased confidence in the findings.

In conclusion, PSE was not a pathway underlying intergenerational transmission of MCM to offspring psychopathology; although PSE was related to greater adolescent‐reported externalizing symptoms. Our findings demonstrate the complexity of intergenerational maltreatment, PSE, and the postnatal caregiving environment, underscoring the importance of evaluating historical risks that mothers may transmit from their own childhood maltreatment and prenatal substance use in assessing offspring psychopathology.

## Ethics statement

Parental written informed consent and child assent have been appropriately obtained. Study procedures were approved by the University Hospitals Institutional Review Board (UHCMC 10‐89‐206; the date of approval 05/26/2006).

**Key points**

- What's known: The impact of childhood maltreatment may cut across generations, extending its long‐term implications. Few studies examined the intergenerational impact of maternal childhood maltreatment in the context of prenatal substance use.
- What's new: Both maternal childhood maltreatment and prenatal substance use increased offspring vulnerability to psychopathology at age 12 directly and indirectly in a low‐income urban sample. Maternal prenatal substance use was not a pathway underlying intergenerational transmission of childhood maltreatment; although maternal prenatal substance use was related to greater adolescent‐reported externalizing symptoms.
- What's relevant: The current study highlights the importance of evaluating historical risks that mothers may transmit from their own childhood maltreatment and prenatal substance use when assessing offspring psychopathology.

## Data Availability

The data that support the findings of this study are available on request from the corresponding author. The data are not publicly available due to privacy or ethical restrictions.
